# Zinc doped BiOBr impregnated into PVDF sponge as a dip-Photocatalyst for RhB removal from wastewater

**DOI:** 10.1038/s41598-025-26948-4

**Published:** 2025-11-28

**Authors:** Mohamed Ahmed, Alaa Farid, Hassan Nageh, Gehan Safwat, Fayrouz S. Mohamed, Mohamed Taha

**Affiliations:** 1 Faculty of Biotechnology, October University for Modern Sciences and Arts (MSA), Giza, 12566 Egypt; 2 Nano Gate Company, 9254 Hoda shaarawy, Al Abageyah, El Mukkatam, Cairo, 11571 Egypt; 3 Central Metallurgical Research and Development Institute (CMRDI),, Cairo, 11421 Egypt; 4 Nanotechnology Research Centre (NTRC), The British University in Egypt (BUE), El-Sherouk City, Cairo, 11837 Egypt

**Keywords:** Environmental sciences, Materials science, Nanoscience and technology

## Abstract

Organic contamination of water has sparked concerns since it has an adverse impact on both human health and the ecosystem as a whole. In this study, Bismuth Oxybromide (BiOBr) was prepared via a Solvothermal approach. Subsequently, BiOBr was doped with Zinc metal to improve the photocatalytic activity through introduce Oxygen vacancies (O_Vs_). The as-prepared materials were characterized using various techniques; Field-Emission scanning electron microscopy (FE-SEM) & Energy dispersive X-ray (EDAX) and elemental composition analysis, X-Ray diffraction (XRD), FTIR spectroscopy and X-ray Photoelectron spectroscopy (XPS). Additionally, optical features (Optical Absorption, band gab, and PL Spectroscopy) and electrochemical impedance spectroscopy (EIS) were also evaluated. The FE-SEM confirmed that, the formation of BiOBr and Zn-BiOBr in a hierarchical microspheres structure constructed from nano-leaves. The physico-chemical characterizations confirm the generation of O_Vs_ upon doping with zinc, the optical features results showed a slight increase in optical band gab of BiOBr (2.811 eV) upon doping with Zinc (2.831 eV) while, the PL of BiOBr is higher than that of Zn-BiBOr and EIS results confirms the lower resistance of charge transfer of Zn-BiBOr indicating electron–hole separation leading to improvement and enhancement RhB’s degradation efficiency, with achieving 100% removal after 35 min of reaction. However, the challenge of photocatalyst (as a suspension) separation after the degradation reaction remained. To resolve this issue, we developed a simple technique to impregnate Zn-BiOBr into a highly porous sponge based on Polyvinylidene Fluoride polymer (PVDF) as a dip-photocatalyst, offering potential as a re-usable photocatalyst matrix. Furthermore, the 3D Zn-BiOBr photocatalyst sponge was tested and Found to sustain up to five cycles in consecutive cycles with almost the same photocatalytic effectiveness. In conclusion, the PVDF − Zn-BiOBr sponge is a promising material for energy conversion applications and environmental purposes and enables the reuse of the photocatalyst several times easily.

## Introduction

Industrialization has brought about both benefits and environmental challenges, notably in the area of energy shortage and water pollution stemming from various pollutants^1^. Wastewater containing pharmaceuticals^2^, heavy metals^3,4^, and dyes^5–7^ poses significant risks to the environment and human health^8^. Traditional wastewater treatment methods fall short in addressing emerging pollutants, necessitating more advanced technologies^9–11^. Efforts to eliminate stable organic dyes from industrial wastewater involve different methods such as adsorption^9,12,13^, coagulation, and semiconductor-based photocatalytic degradation^8,14,15^, with the latter gaining attention for its appealing qualities, such as the use of environmentally friendly materials and straightforward operational procedures leading to efficient degradation^1,16^. Photocatalysis emerges as a promising solution that utilizing sunlight to break down organic pollutants^2,5,17–19^, hydrogen production and photocatalytic hydrogenation of pharmaceutical compounds^20^. Recent studies focus on enhancing the photocatalytic activities properties of semiconductors for energy conversion applications^21^, organic synthesis^22^, and improve their photocatalytic activities through heterojunction formation^23–25^ or incorporation of heteroatom to enhance oxygen defect for improving organic’s degradation^26^. Bismuth Oxybromide (BiOBr) has gained prominence as a photocatalyst for degrading organic pollutants^27^. Due to its stability and high photocatalytic performance, BiOBr prepared in various structures such as nanospheres, nanosheets, and nanotubes, with study its photocatalytic features in applications such as; hydrogen evolution and organic substance degradation. The tetragonal sphalerite structure of BiOBr, featuring bilayers of [Bi_2_O_2_] sheets and double slabs of Br atoms, creates an electrostatic field promoting the separation of photo-generated charges, enhancing overall efficiency as a photocatalyst. However, a significant drawback is the high rate of electron-hole recombination in BiOBr, limiting its practical utility. Addressing challenges related to reducing recombination and shifting the absorption edge toward the visible spectrum is crucial for enhancing the photocatalytic performance of BiOBr. To enhance the photocatalytic efficiency and responsiveness to visible light of BiOBr, researchers have employed strategies such as ion doping^28^, creating heterojunctions^1,3,14,29–31^, introducing oxygen vacancies^10^, and modifying with noble metals. Heterostructure formation by conjugation with other semiconductor to enhance photocatalytic performance have been reported, including (BiOBr-g-C_3_N_4_^1,32–34^, BiOCl-BiOBr^35,36^, BiOBr–ZnFe_2_O_4_^37,38^, BiOBr–TiO_2_^39^, CdS/BiOBr^40^, and MoS_2_-BiOBr^41^ were synthesized and all of which displayed enhanced photocatalytic efficiencies.

In the pursuit of enhanced photocatalytic performance, 3 d transition metals (TMs) and non-metals have been introduced through doping. Notably, metals such as Cu, Zn, Ni, Ag, Al, Fe, and Mn act as a capture centers of electrons, enhancing effective separation of photo-generated charges and improving BiOBr’s photocatalytic activity^9,29,42,43^. Zn doping specifically offers a promising avenue for enhancing the photocatalytic activity of BiOBr^15,44^. Incorporating zinc atoms into the BiOBr crystal lattice influences its electronic structure, potentially altering the band gap, a crucial parameter determining light absorption. Zn doping aims to narrow the band gap, making BiOBr more responsive to visible light and enhancing its ability to generate electron-hole pairs under illumination^16,44,45^. This modification holds potential for advancing the overall efficiency of BiOBr as a photocatalyst, especially in a potential application candidate for photocatalytic water splitting and photocatalytic degradation of organic pollutants under irradiation of visible light depending on its suitable energy band gap, stable Physico-chemical features and high productivity^15^.

To enhance the recyclability and reusability of photcatalysts, the active photocatalysts were incorporated into PVDF polymer membrane. Excellent photocatalytic activity and a photocatalytic degradation rate of 91.84% after 270 min were demonstrated by the SnO_2_/TiO_2_/PVDF composite membrane when the nanocatalyst content was 7% w/w of the PVDF matrix^46^. Additionally, it was found that the PVDF incorporated with heterogeneous photocatalysts had outstanding recyclability, long-term activity, and thermal stability^47^. A feasible approach for wastewater remediation is the production of eco-friendly hydrogel materials of nanostructured CoFe_2_O_4_ (CF) particles embedded in a hydrogel matrix constructed from carboxymethyl cellulose–psyllium–RGO (CPRHG/CF) was used for removal of pharmaceutical effluent^48^ additionally, fabrication of CMC/PVP-RGO-NiFe_2_O_4_ (CPRN) based hydrogel was used successfully for the adsorption and photo reduction removal of hexavalent chromium (Cr VI)^49^.

Floating porous Polydimethylsiloxane (PDMS) sponge based-photocatalyst has offered a feasible and promising technology for efficient organic pollutants’ removal from the environment and energy conversion’s applications^50–55^ and photocatalytic hydrogenation of p-nitrophenol in continuous flow system^56^. It can be easily and eco-friendly fabricated through a soluble salt or sugar cube template method^52,57^. Typically, this kind of catalyst is referred to as a “dip-catalyst.”^55,58,59^, Since PDMS and PVDF are commercially available, it is a viable matrix for creating dip-catalysts. In continuous flow processes, where these catalysts attain optimum recyclability, an automated process is feasible.

In this study, through a swift and direct solvothermal technique, we effectively created hierarchical nanostructures of BiOBr and Zn-BiOBr as a visible-light photocatalysts and incorporated into PVDF matrix to form re-usable photocatalyst sponge. The crystal structure, was conducted by XRD investigation, the electronic structure and chemical state by XPS technique. FTIR spectroscopy to identify the chemical bond and function groups. Size & surface Morphology, and elemental composition was monitored via SEM & EDAX & Mapping. Additionally, the optical parameters; optical bandgap (Eg), PL, and EIS investigations were evaluated Finally, the photocatalytic effectiveness of the fabricated materials was assessed through the photocatalytic degradation of Rhodamine B (RhB) under visible light conditions (λ = 420 nm).

## Experimental works

### Raw materials

Cetyl trimethyl ammonium Bromide (CTAB), Ethane-1, 2-diol (EG, C_2_H_6_O_2_), Bismuth Nitrate Pentahydrate (Bi(NO₃)₃·5 H₂O), and Zinc Acetate dihydrate (Zn(CH₃CO₂)₂·2 H₂O) were all procured from Loba-Chemie (India). Absolute Ethanol, Iso-Propyl Alcohol (IPA), Formic Acid (FA), L-Ascorbic acid, and anhydrous sodium sulfate (Na_2_SO_4_) were supplied from Merck-Germany. The polymeric matrix was prepared using Polyvinylidene Fluoride polymer (PVDF; Molecular Weight 270,000 g/mol) and Perfluorinated resin solution containing Nafion™ 1100 W were supplied from Sigma-Aldrich (USA), glacial acetic acid purchased from Chem-Lab Supplies (Belgium).

### Preparation of pristine BiOBr and Zn-Doped BiOBr photocatalyst

Pristine BiOBr and Zn-doped BiOBr (Zn-BiOBr) were synthesized using a one-pot solvothermal approach^60^. Briefly, a 0.05 N solution of CTAB in Ethane-1, 2-diol was prepared to serve as a template and a source of Br⁻ ions. Bi(NO₃)·5 H₂O was dissolved in the CTAB solution in a stoichiometric ratio, ensuring a 1:1 molar ratio between Bi⁺³ and Br⁻. The solution was magnetically agitated for 30–60 min. at normal room conditions to ensure homogeneity. The resulting mixture was then transferred into a Teflon-lined stainless steel autoclave and subjected to a solvothermal treatment at 160 °C for 12 h. After the reaction, the autoclave was allowed to cool naturally to room temperature. The obtained product was separated by centrifugation at 10,000 rpm for 20 min. (Hermle Z32HK, Germany), followed by several washings with double distilled water (DDH_2_O) and absolute ethanol to remove impurities. Finally, the purified materials were dried at 65 °C for 12 h. The synthesis followed a similar protocol with an additional step. Zinc acetate was introduced into the reaction mixture prior to the solvothermal treatment, maintaining a Zn/Bi molar ratio of 1:10 The Zn-BiOBr product was similarly washed, centrifuged, and dried to yield the ZnB photocatalyst.

### Fabrication of 3D Zn-BiOBr sponge

**Fig. 1:**
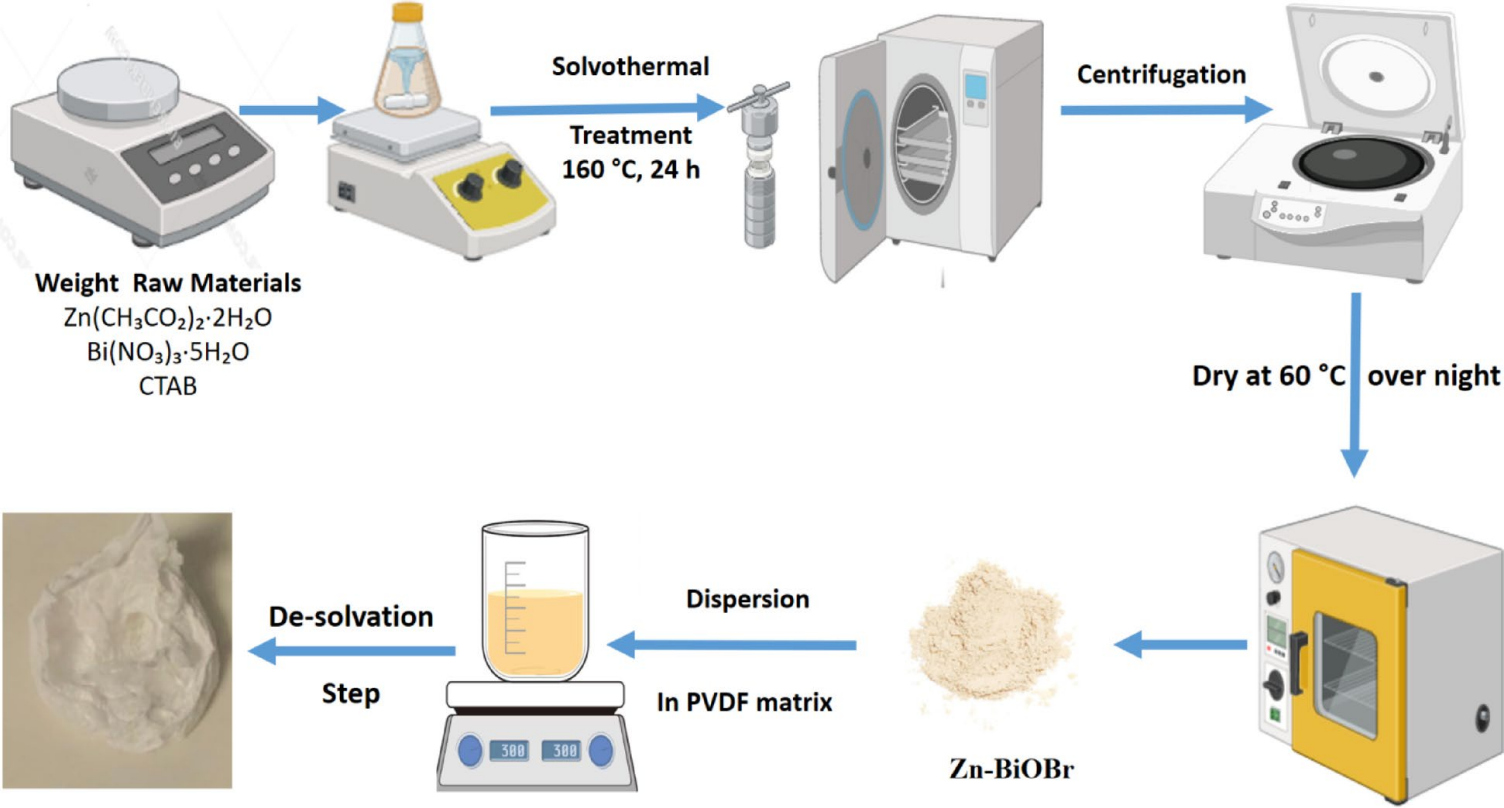
The Schematic diagram of fabrication process.

To fabricate a 3D Zn-BiOBr sponge as a dip-photocatalyst using the De-solvation method, begin by dissolving 10% w/v PVDF polymer in 90% w/v glacial acetic acid under continuous stirring. Once fully dissolved, introduce Zn-BiOBr as a filler at varying concentrations (0%, 10%, and 20% w/w). The mixture is stirred on a hot plate at 90 °C for 2 h to ensure homogeneity. Upon achieving a uniform solution, initiate the desolvation process by adding distilled water, resulting in the formation of a 3D porous sponge structure (Fig. 1). Since the PVDF polymer is insoluble in water, the matrix containing the photocatalyst was de-dissolved and moved to a porous sponge when DH_2_O was added. This method ensures consistent incorporation of fillers and promotes the development of a robust nano-sponge. For ease of identification and handling, the samples were labeled as follows: **ZnBS-0** for the PVDF sponge with 0% Zn-BiOBr (control sample), **ZnBS-10** for the PVDF sponge with 10% Zn-doped BiOBr, and **ZnBS-20** for the PVDF sponge with 20% Zn-BiOBr.

### Physico-chemical characterization

The as-prepared materials were characterized using different techniques. The surface morphology was examined using a Field Emission Scanning Electron Microscopy (FE-SEM, Quattro S, Thermo Scientific), the elemental composition, and distribution of the materials were determined using Energy Dispersive X-ray (EDAX) and elemental mapping. The crystalline phase of the synthesized materials was investigated via XRD using an Empyrean Malvern Panalytical (Netherlands) instrument, with 2θ ranging from 5° to 90°, a step size of 0.04°, and a Cu Kα radiation wavelength of 1.5406 Å. The electronic structure and chemical state of the photocatalysts were investigated using XPS technique on an ESCALAB 250Xi (Thermo Fisher, USA). Attenuated total reflectance Fourier Transform Infrared (ATR-FTIR) spectra were recorded at room temperature (RT) using a Vertex 70-RA M II (Bruker Analytical, Germany) over a spectral range of 400–4000 cm⁻¹. The JASCO Corp. V-570 UV-Vis spectrophotometer was utilized to determine the UV-Vis diffused reflectance spectrum (UV-Vis-DRS) of photocatalysts. While, the UV-Vis optical absorption spectra were measured with a Cary series UV-Vis-NIR spectrophotometer (Australia) in the wavelength range of 200–800 nm. Additionally, photoluminescence (PL) studies were measured at RT using a Lumina Fluorescence Spectrometer (Thermo Scientific) with an excitation wavelength of 320 nm.

### Electrochemical measurements

The electrochemical characterization was performed in 0.1 M Na_2_SO_4_ electrolyte solution.

The glassy carbon electrode, which has a surface area of 0.071 cm2, is used as the working electrode. After being polished with soft emery paper, it was rinsed with ethyl alcohol and double-distilled water (DD.H_2_O). Then, catalysts were produced by ultrasonically suspending catalyst powder (15 mg) in a mixture of 0.5 mL isopropanol, 0.5 mL DD.H_2_O, and 0.5 mL 5 wt% Nafion solutions for 15 min. After this, 50 µL of ink was applied to the surface of the glassy carbon electrode and left to dry overnight in a desiccator. Autolab PGSTAT128N was utilized to collect data for electrochemical impedance spectroscopy (EIS) using NOVA electrochemistry program, the impedance spectrum was fitted (Version 2.1, Metrohm Autolab, Utrecht, Netherlands). Three electrode cells were built using BiOBr and Zn-BiOBr catalysts as working electrodes, separately, Ag/AgCl/KCl (sat.) and Pt wire as a reference electrode and an auxiliary electrode respectively. A consistent AC voltage value was maintained during EIS tests by applying an AC voltage amplitude of 10 mV and a frequency range of 1 × 10^4^ Hz to 100 mHz. All electrochemical experiments were conducted in de-aerated solutions at ambient temperature.

### The photocatalytic measurement

The photocatalytic measurement of the prepared materials was tested under visible light irradiation using a 40 W lamp (Sylvania, Germany) as an illumination source. In the photodegradation tests, 100 mL of 1 × 10^− 5^ M RhB dye was mixed with 0.1 g of the photocatalyst. After agitating the mixture for an hour in the dark to achieve adsorption-desorption equilibrium, the light is turned on to start the photocatalytic reaction. The photocatalyst was settled and the pure dye was decanted by centrifuging a 3 mL sample every 5 min. To assess the fabricated sponge’s photocatalytic performance; a piece (1 gm) of ZnBS-0, 10, or 20 sponge was placed into 100 mL of 1 × 10^− 5^ M RhB dye with repeating the previous steps. A UV–vis spectrophotometer (Cary series UV-Vis- NIR, Australia) was utilized to evaluate the absorbance of the RhB solution then, using Beer Lambert’s law to determine its corresponding concentration.

### Trapping experiment

Various scavengers were used in the trapping examines in an aqueous solution of RhB. Three scavengers, Isopropyl alcohol, ascorbic acid, and formic acid were added separately to the RhB solution after the catalyst had been added. These scavengers were utilized to capture the hydroxyl radical (^•^OH), superoxide radical (^•^O_2_^−^), and hole (h^+^), respectively^61,62^. All reaction conditions remained the same, and the fixed concentration of all scavengers was 0.001 M, 0.5 mL. After collecting 2 mL aliquots at various times, the photocatalyst particles were eliminated using PTFE Syringe Filters (0.45 μm) and the absorbance of the solution was measured using a UV-vis spectrophotometer (Cary series UV-Vis-NIR, Australia).

### Recyclability test

To investigate the recyclability of Zn-Doped BiOBr sponge, five runs were conducted to assess its effectiveness in RhB degradation. Between each run, the sponge was repeatedly cleaned with deionized water.

## Results & discussions

### Physico-chemical characterization

**Fig. 2:**
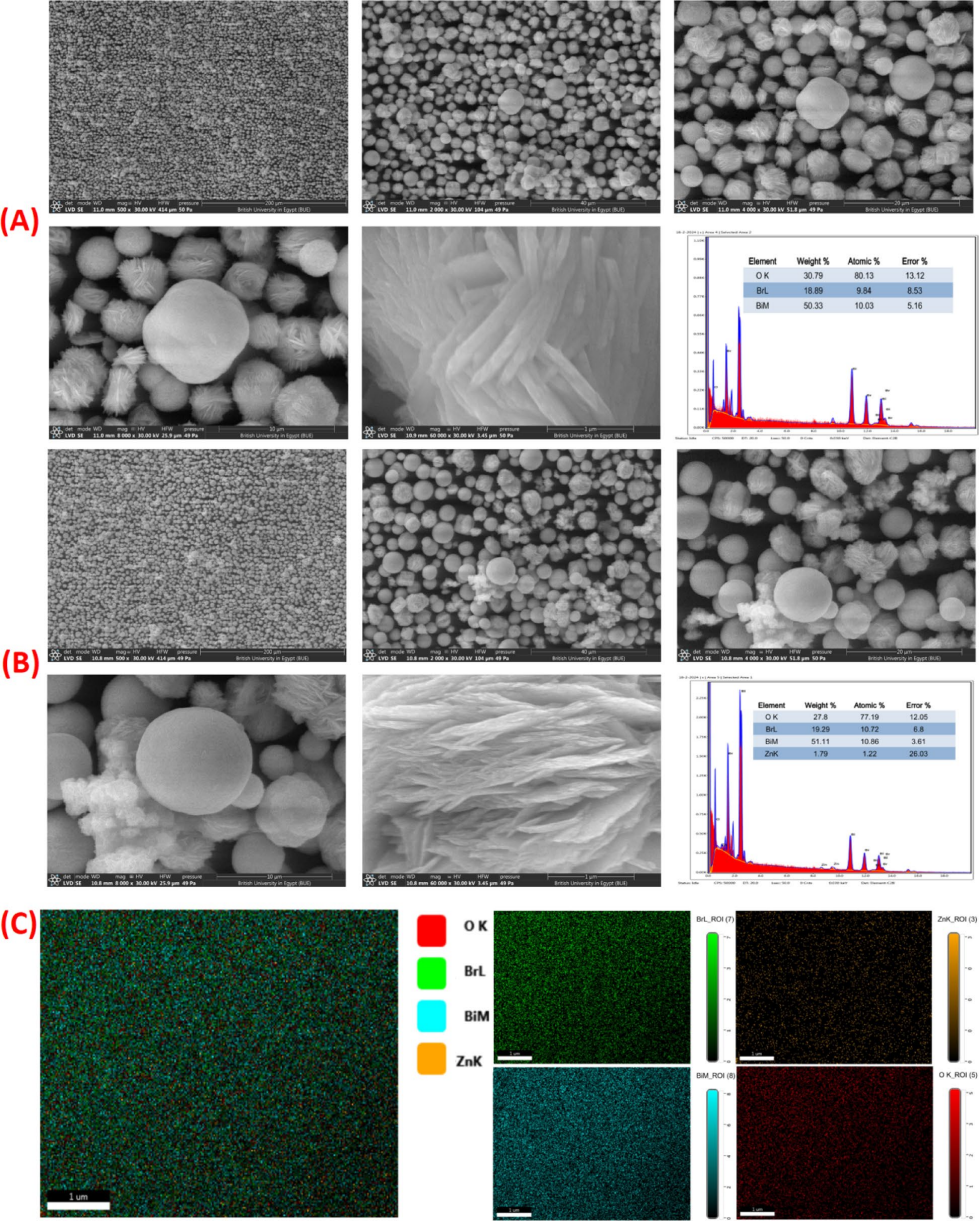
**(A)** The SEM images at different magnifications of BiOBr with corresponding EDAX, **(B)** Zn-BiOBr with corresponding EDX, and **(C)** elemental mapping of Zn-BiOBr microflower respectively.

Figure 2(A-C) depicts the SEM, EDAX, and elemental mapping of BiOBr and Zn-BiOBr respectively. Figure 2A shows the SEM image of pristine BiOBr, exhibits a well-defined hierarchical structure constructed from homogeneously dispersed microspheres. These microspheres fall in the range of ca. 5: 6 μm with an opening and loose packing of nanosheets. the corresponding EDAX spectrum confirms the presence of Bi, O, and Br with a very close weight and atomic percentages to the theoretical stoichiometry of BiOBr which confirm the high uniformity and purity of pristine BiOBr. Figure 2B presents the SEM image of Zn-BiOBr, showing obvious morphological variation compared to the pristine BiOBr. Both samples exhibit hierarchical microspheres constructed from nano-leaves; however, the microspheres of Zn-BiOBr are in an average diameter of about 5: 6 μm, and are made up of nanosheets with thin nano-leaf structures (~ 70 ± 10 nm), The EDAX corresponding spectrum confirms that Zn has been successfully incorporated into the BiOBr. The elemental mapping of Zn-BiOBr (Fig. 2C) results confirmed the successful integration of zinc into the BiOBr matrix is suggested by the fact that all of the components are uniformly distributed throughout the scanned region and that there is no indication of any agglomeration or segregated phases in the mapping of the composite.

**Fig. 3:**
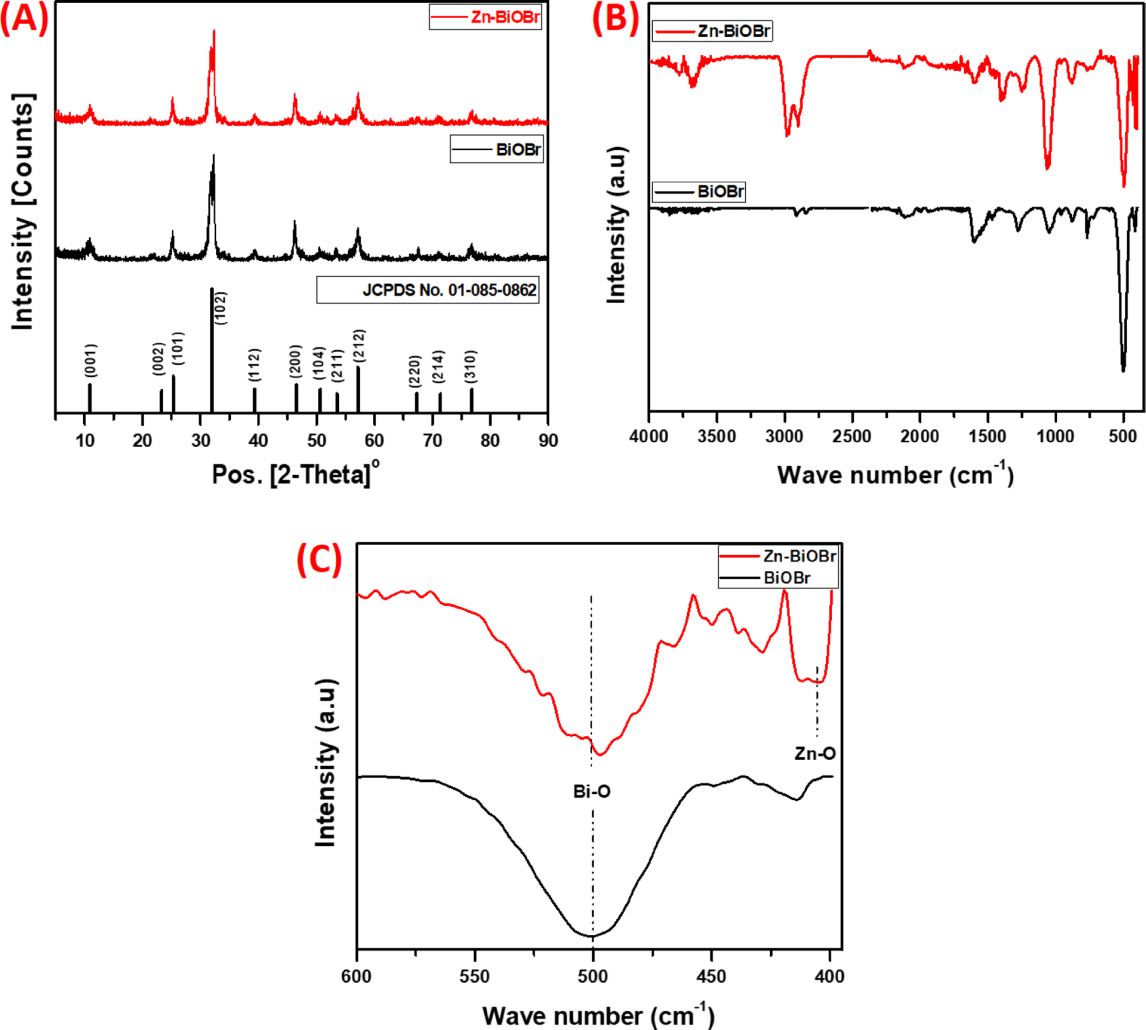
**(A)** The X-ray diffraction pattern, **(B)** The FTIR spectra in the range 4000:400 cm^− 1^, and **(C)** The FTIR spectra in the range 600:400 cm^− 1^ of BiOBr and Zn-BiOBr respectively.

The crystalline structures and the impact of Zn doping are shown by the X-ray diffraction (XRD) patterns (Fig. 3A.) of pristine BiOBr and Zn-BiOBr. A significant degree of crystallinity is indicated by distinct and sharp diffraction peaks seen in both samples. According to JCPDS No. 01–085-0862, these peaks correspond to the tetragonal layered structure of BiOBr, as seen in Fig. 3A with prominent reflections indexed to the (001), (002), (101), (102), (110), (112), (200), (104), (211), (212), (220), (214), and (310) planes. In accordance with the standard pattern of BiOBr, the equivalent 2θ values are 10.91°, 21.63°, 25.21°, 31.73°, 32.31°, 39.31°, 46.37°, 57.21°, 67.46°, 71.00°, and 76.76° respectively. In the case of pristine BiOBr, intense diffraction peaks are observed, particularly for the (102) and (110) planes, indicating a preferential orientation of the crystallites along these directions, which is a characteristic feature of its layered structure, as seen in Fig. 3A^63^. For Zn-BiOBr, the primary diffraction peaks remain consistent with those of pristine BiOBr, confirming that the tetragonal phase is preserved after Zn incorporation as well as confirms that Zn is homogeneously doped into the BiOBr lattice without forming separate crystalline phases. Nonetheless, slight shifts in peak positions and variations in intensity, particularly in the (102), (110), and (200) planes, are observed. These shifts indicated that Zn²⁺ ions, with a smaller ionic radius (0.6 Å) compared to Bi³⁺ (0.96 Å), are successfully doped into the BiOBr lattice by substituting Bi³⁺ ions^15,64^. The lattice distortions and variation in the intensity and broadening of certain peaks caused by Zn doping lead to modification of the electronic structure of the material through enhanced charge carrier mobility and creation of defect states. These defect states favor effective charge separation while reducing recombination, thus enhancing photocatalytic activity^44,65^.

The FTIR study was conducted to investigate the structural and chemical bonding modifications induced by Zn doping into BiOBr crystal structure. Figure 3B; presented the FTIR spectra of BiOBr and Zn-BiOBr in range 4000 cm^− 1^: 400 cm^− 1^, the both samples have major peaks; the broad band in the range 3744 cm^− 1^ – 3611 cm^− 1^ in attributed to the stretching vibration of O-H coordinated with Bi^3+^ showing the presence of moisture^60,66^. The bands within the range of 1500 cm^− 1^ – 1000 cm^− 1^ correspond to the Bi-Br bond in BiOBr^18^. The strong absorption peak at 501 cm^− 1^ is assigned to the Bi-O stretching vibration mode^30^. A small peak was appeared at 1390 cm^− 1^ in Zn-BiOBr representing the oxygen vacancy (O_V_) vibration, indicating that Zn doping provided more favorable conditions for O_V_ formation^67,68^. Furthermore, a noticeable reduction in the intensity of the Bi-O stretching vibration at 550 cm^− 1^ and appearance of a new peak at 450 cm^− 1^, that assigned to Zn-O starching vibrations, unequivocally confirms the successful substitution of Bi^3+^ ions with Zn^2+^ ions in the BiOBr lattice (Fig. 3C)^60^.

**Fig. 4:**
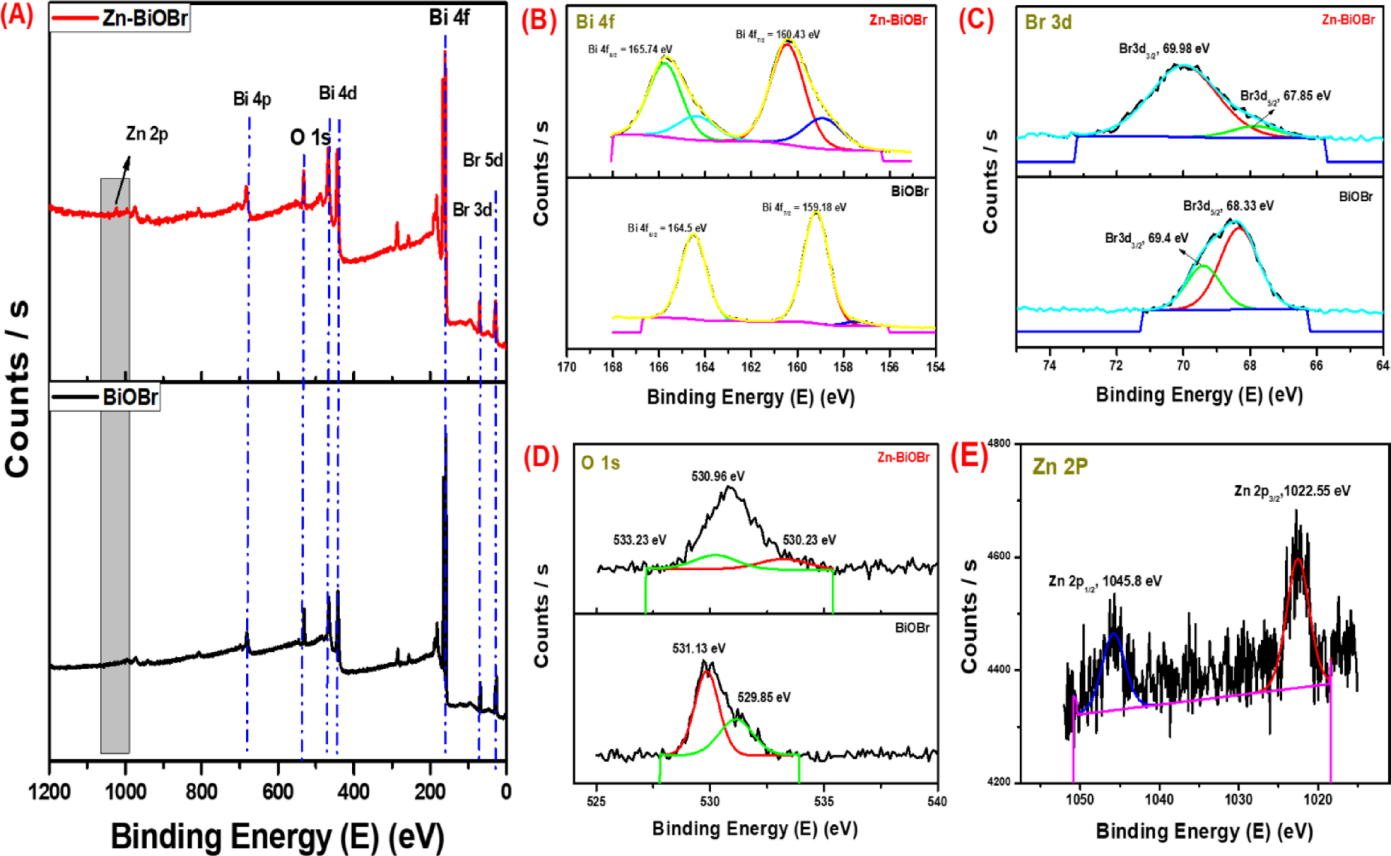
**(A)** The XPS survey spectra, **(B**,** C**, and **D)** the high resolution XPS spectra (Bi 4f, Br 3d, and O 1s) of BiOBr and Zn-BiOBr respectively, and (E) high resolution XPS spectrum of Zn 2P of Zn-BiOBr.

The elemental composition and chemical state of BiOBr and Zn-BiOBr were examined using the XPS Spectra. Figure 4A shows The survey spectra of BiOBr and Zn-BiOBr displayed the main peaks of Bi, O, and Br meanwhile, the peak of Zn was observed in Zn-BiOBr indicated successful doping of Zinc into BiOBr structure. The Bi 4f high resolution XPS spectra **(**Fig. 4B**)** can be fitted into two main peaks corresponding to Bi 4f_7/2_ and Bi 4f_5/2_ and located at 159.18 eV and 164.5 eV for BiOBr while, blue-shifted in Zn-BiOBr to 160.44 eV and 165.75 eV respectively. As illustrated in Figure 4C, two peaks representing Br 3d_5/2_ and Br 3d_3/2_ have been determined from the Br 3d spectra at (68.33 eV and 69.4 eV) for BiOBr and (67.85 eV and 69.98 eV) for Zn-BiOBr, respectively^20,52,69^. The high resolution XPS of O1s **(**Fig. 4D**)** of BiOBr is composed of two main peaks at 531.13 eV and 529.85 eV representing the lattice oxygen, and oxygen species conjugated on the surface such as OH groups. meanwhile, the O1s of Zn-BiOBr spectrum is composed of three main peaks localized at: 533.23 eV, 530.96 eV and 530.23 eV for Zn-BiOBr. The peak at 533.23 eV is attributed to oxygen species conjugated on the surface such as OH groups. Furthermore, the peak at 530.96 eV is assigned to the Bi-O bond of BiOBr, while, the peak at 530.23 eV is reasonably corresponding to the oxygen vacancies generated upon doping with Zinc^69,70^. For all shared elements; a slight peak’s blue shift was found in Zn-BiOBr compared with BiOBr, suggesting that Zn doping caused a change in the chemical environment of Bi, which was correlated with a change in local chemical bonds due to Zn doping. Higher binding energy means less electrons attaching on corresponding chemical bond, which is usually correlated to forming of trapping center like atom vacancy or lattice defect^68^. Combined with O_V_ peak in FT-IR spectra **(**Fig. 3B**)**, O_V_ formation and its trapping impact in the lattice might be indirectly confirmed by the blue shift of binding energy in Zn-BiOBr.

From the Zn 2P spectrum of Zn-BiOBr, as illustrated in Fig. 4E, two peaks of Zn 2P_3/2_ and Zn 2P_1/2_ were extracted at 1022.55 eV and 1045.80 eV, respectively^15^.

### Optical, electrochemical properties, and energy band structure

**Fig. 5:**
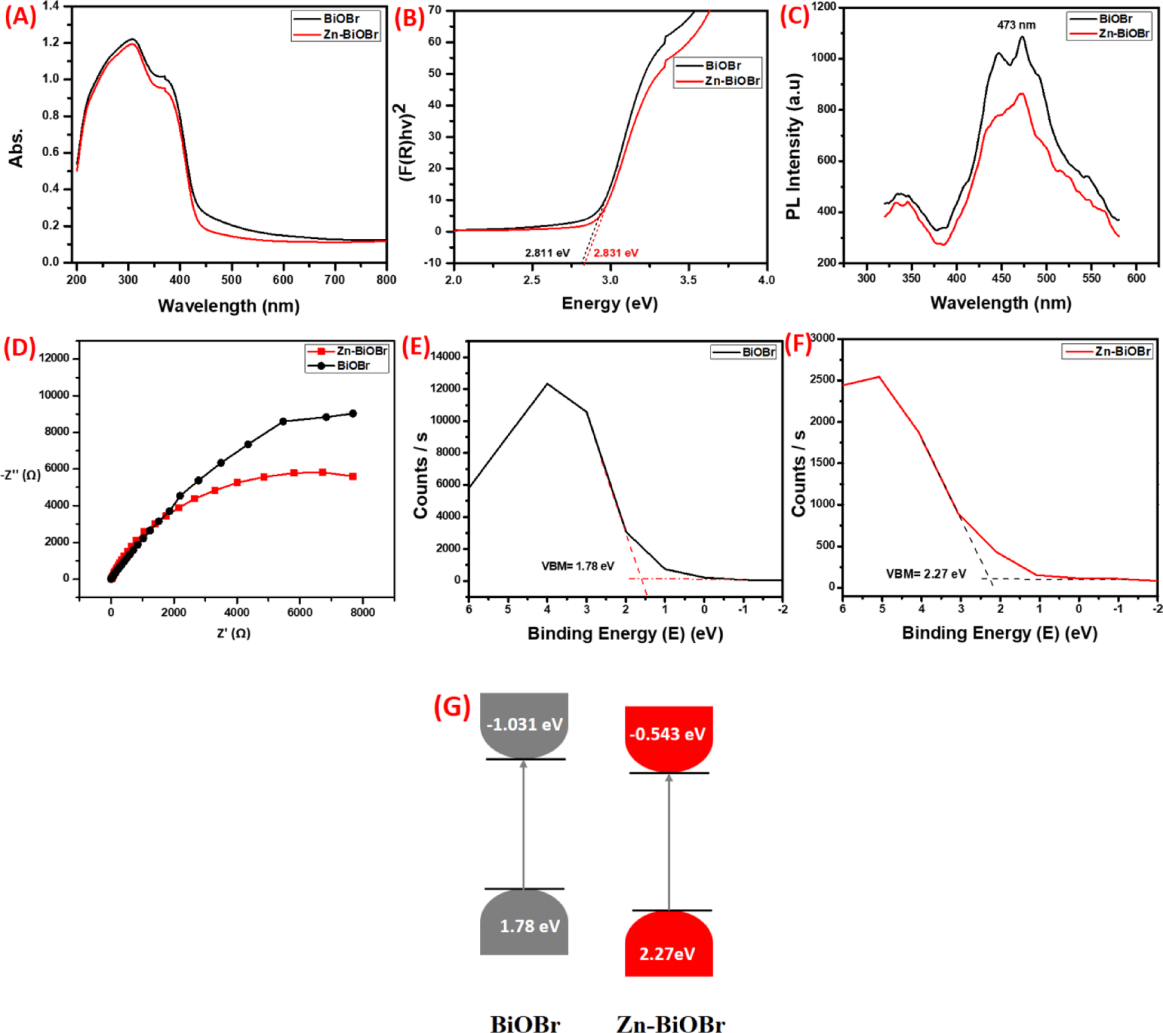
**(A)** The absorption spectra, **(B)** The plot of (αhυ)^2^ vs. hυ, **(C)** PL spectra, and **(D)** EIS Nyquist spectra under 0.1 M Na_2_SO_4_ solution of BiOBr and Zn-BiOBr respectively, XPS valence band of **(E)** BiOBr, and **(F)** Zn-BiOBr, and **(G)** energy band diagrams.

The absorption spectrum of BiOBr and Zn-BiOBr is distinguished by a significant absorption at a maximum wavelength of approximately 308 nm (Fig. 5 A)^60^. Tauc’s relation between (αhυ)^2^ and the incident photon energy hν was plotted using the absorption data as follows:

1 $$\:{\left(\alpha\:h\upsilon\:\right)}^{2}=constant\:(h\upsilon\:-{E}_{g})$$

where α is the absorption co-efficient and E_g_ is the direct bandgap energy^21,71^. As showed in Fig. 5B, the BiOBr displayed E_g_ value about 2.811 eV while, 2.831 for Zn-BiOBr. Furthermore, the PL emission of the obtained samples is shown in the range of 350–600 nm using an excitation wavelength of λ = 320 nm, as presented in Fig. 5C. In brief, pure BiOBr exhibits the highest PL emission, centered at approximately 473 nm, which corresponds to the high recombination rate of photo-generated charges. In contrast, the Zn-BiOBr sample shows the lowest PL intensity, demonstrating a notable decrease in electron-hole recombination upon Zn doping, due to faster carrier transportation with O_V_ trapping leading to enhanced carrier separation in Zn-BiOBr subsequently, enhancement of the photocatalytic activity of the material^60,66^. Related photoluminescence (PL) and electrochemical test were carried out in order to more specifically assess the modulation produced by Zn doping on carrier transportation behavior modulation and its correlation with photocatalytic performance. As shown in Fig. 5D, Zn-BiOBr had a smaller arc radius than BiOBr, which indicated that carriers had less resistance on Zn-BiOBr confirming faster electron-hole separation as a result of O_V_ trapping due to, providing more favorable condition for O_V_ generation upon doping with zinc as suggested by Guan, Chongshang, et al.^68^. More photo-generated carriers were able to participate in the reaction process due to faster carrier separation and transfer, increasing the yield of the final product^61,62,68^.

Furthermore, to explain the mechanism of photocatalytic activity, it is necessary to confirm the valence band (VB) and conduction band (CB) potentials of BiOBr and Zn-BiOBr. As shown in **(**Fig. 5E, F**)** the valence band edges were obtained by VB-XPS spectra. It is observed that the VB potentials of the BiOBr and Zn-BiOBr are at 1.78 and 2.27 eV. Based on the results of DRS, the band edge positions of the conduction band (CB) of BiOBr and Zn-BiOBr can be calculated based on the following Eqs^72,73^.

2 $$\:ECB=\:EVB-\:\:Eg$$

According to the results of VB and band gap energies (E_g_) of BiOBr and Zn-BiOBr, band diagrams emerged as seen in Fig. 5G.

### Photocatalytic measurements

**Fig. 6:**
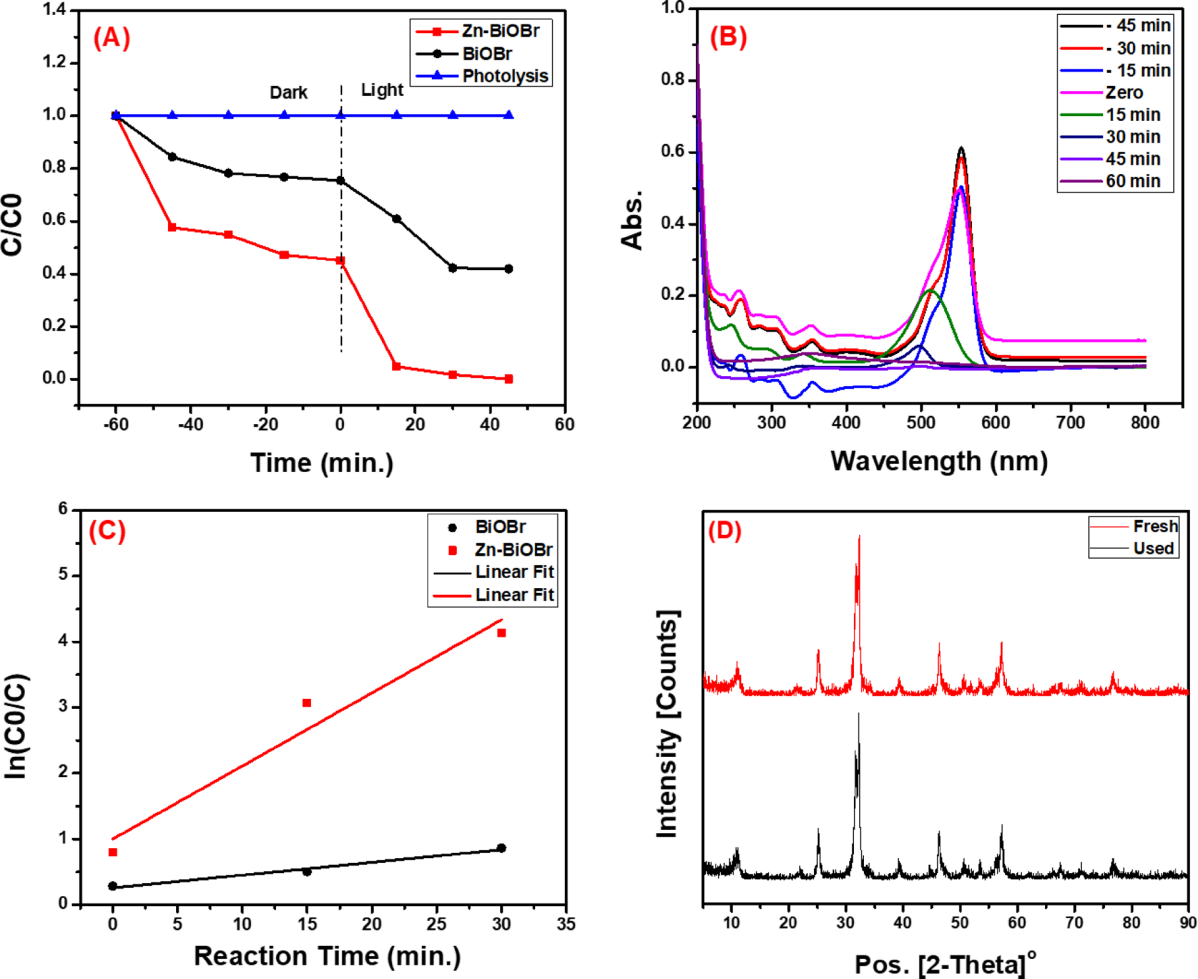
(**A**) The photocatalytic degradation of RhB **B**) UV-Vis spectra of RhB’s degradation using Zn-BiOBr, (**C**) the rate constant of the BiOBr and Zn-BiOBr photocatalyst, and (D) XRD pattern of fresh and used Zn-BiOBr catalyst.

The photocatalytic activity of the as-prepared materials was evaluated by monitoring the degradation of RhB dye under visible light irradiation. Figure 6A shows RhB’s photo-catalytic degradation using BiOBr and Zn-BiOBr photocatalysts of the same weight. As illustrated, by doping BiOBr with Zinc, the photocatalytic degradation of RhB dye after 35 min increased from 60% to 100%. This doping technique had a major impact on their photocatalytic performance, these results were consistent with earlier studies^15,45,74^. Furthermore, the Fig. 6B depicts the change of absorption spectra of RhB by Zn-BiOBr at different interval time. In general, doped Zn atom stimulated strong synergistic effect between adjacent O_V_. Zn doping firstly modified energy band structure of BiOBr^15,68^, rendering highly dispersive energy band, beneficial to faster carrier transportation. Secondly, doping induced lattice distortion excited O_V_ generation, which worked as trap center, separating carriers assisted by fiercer covalent unsaturation due to Zn doping, enhancing carrier separation ability. Thirdly, corresponding intermediate energy level due to O_V_ introduction strengthened band to band transition, leading to synergy effect between Zn and O_V_ sites. Furthermore, doped Zn atom would synergize with induced O_V_ and collectively lower energy barrier for RhB degradation. It could be concluded that Zn doping brought about crucial reason for prominent promotion of photocatalytic performance. Therefore, enhancing photocatalytic performance would not be mainly driven by a lower bandgap or more noticeable absorbance^68^. Additionally, Fig. 6C shows the linear relationship between ln(C_0_/C) and time. The corresponding reaction kinetic rate constant (k) and regression coefficients (R^2^) were calculated and listed in Table 1. The obtained results showed that the RhB’s degradation reaction proceeded with first-order kinetics. As illustrated in Table 1, the kinetic rate constants of BiOBr and Zn-BiOBr are 0.0194 ± 0.00291 min^− 1^, and 0.111 ± 0.0233 min^− 1^, respectively. It’s clear that, the rate constant of Zn-BiOBr is higher than that of BiOBr. Furthermore, The XRD was used to study changes in patterns after the photocatalytic re-use (Fig. 6D) The patterns show slight variations between the fresh material’s and the used catalyst’s peak intensities, indicating the Zn-BiOBr catalyst’s reasonable stability.

**Table 1:** The kinetic rate (k) and regression coefficients (R^2^) of BiOBr and Zn-BiOBr.

No.	Photocatalyst	K (min^−1^)	R^2^
1	**BiOBr**	0.0194 ± 0.00291	0.955
2	**Zn-BiOBr**	0.111 ± 0.0233	0.915

**Fig. 7:**
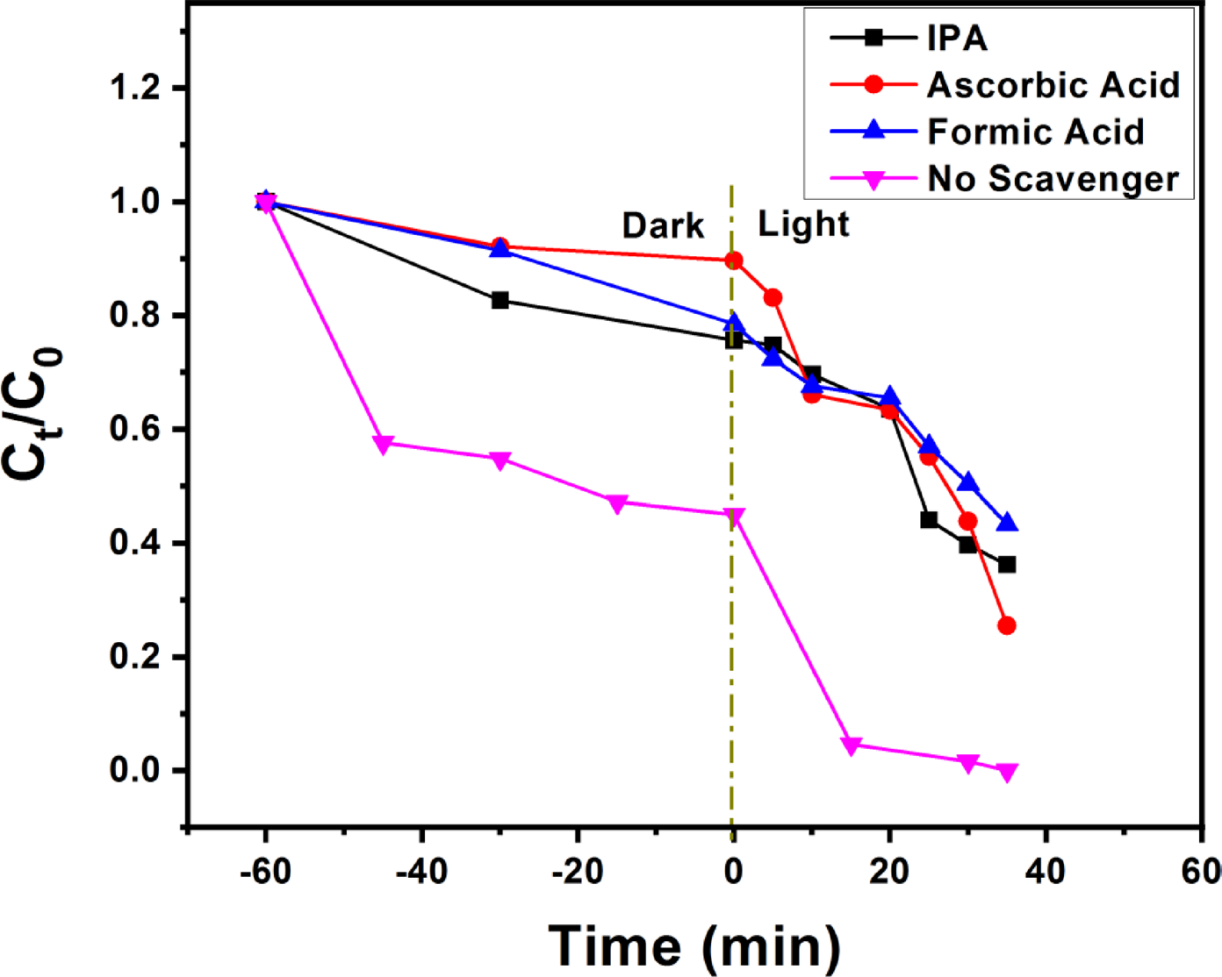
The effect of different scavengers (IPA, AA, FA, and without scavenger) on RhB degradation using Zn-BiOBr.

Based on the trapping experiments, the photocatalytic mechanism of RhB degradation on the surface of Zn-BiOBr with enhanced oxygen vacancies was proposed as shown in Fig. 7. Comparatively, photocatalytic activities are observed to be greatly increased when either ^•^O_2_^−^ scavenger (75%, AA) or ^•^OH scavenger (64%, IPA) is added to the corresponding RhB photodegradation system. Based on the XPS-VB results (Fig. 5F), the potentials of conduction and valence bands of Zn-BiOBr were found to be −1.83 and 0.98 eV respectively. When irradiated with appropriate photons, photo-generated carriers were formed. The holes have more positive and oxidative potential and oxidize H_2_O (H_2_O/^•^OH = 2.38 eV vs. NHE) or OH^−^ (OH/^•^OH = 1.99 eV vs. NHE) into ^•^OH radicals which took part direct part by degrading the RhB pollutant. Because scavengers quickly consume ^•^OH, which increases the availability of photogenerated h^+^, ^•^OH inhibit RhB photodegradation. The AA and IPA quenching studies further suggest that photogenerated holes might be rate-limiting species. When FA (h^+^ scavenger) was added, RhB degrading activity dramatically decreased (57%), confirming the essential rate-determining roles of h^+^^75^. In the photocatalytic system, ^•^O_2_^−^ should be related to the photogenerated electrons at CBMs of Zn-BiOBr because its CBMs (−0.543 eV) is more negative than ^•^O_2_^−^ formation energy (O_2_/^•^O_2_^−^ = − 0.33 eV)^15^. The photogenerated electrons at CBM can be injected from both excited RhB (photosensitization effects) and excited photocatalysts, given that RhB has a higher negative LUMO (−1.77 eV vs. RHE) than CBMs^76^. But because AA can quench ^•^O_2_^−^ in the RhB photodegradation reaction, which limits recombination and increases VBM h^+^, AA increased RhB photodegradation was observed. The induced O_V_ also capture electrons which contribute to the reduction of O_2_ to generate ^•^O_2_^−^ radicals. The O_V_ are reported to uplift oxygen adsorption and the chemisorption can weaken O–O bond thus enhancing reduction of O_2_ to ^•^O_2_^−^ radicals^26^.

**Scheme 1:**
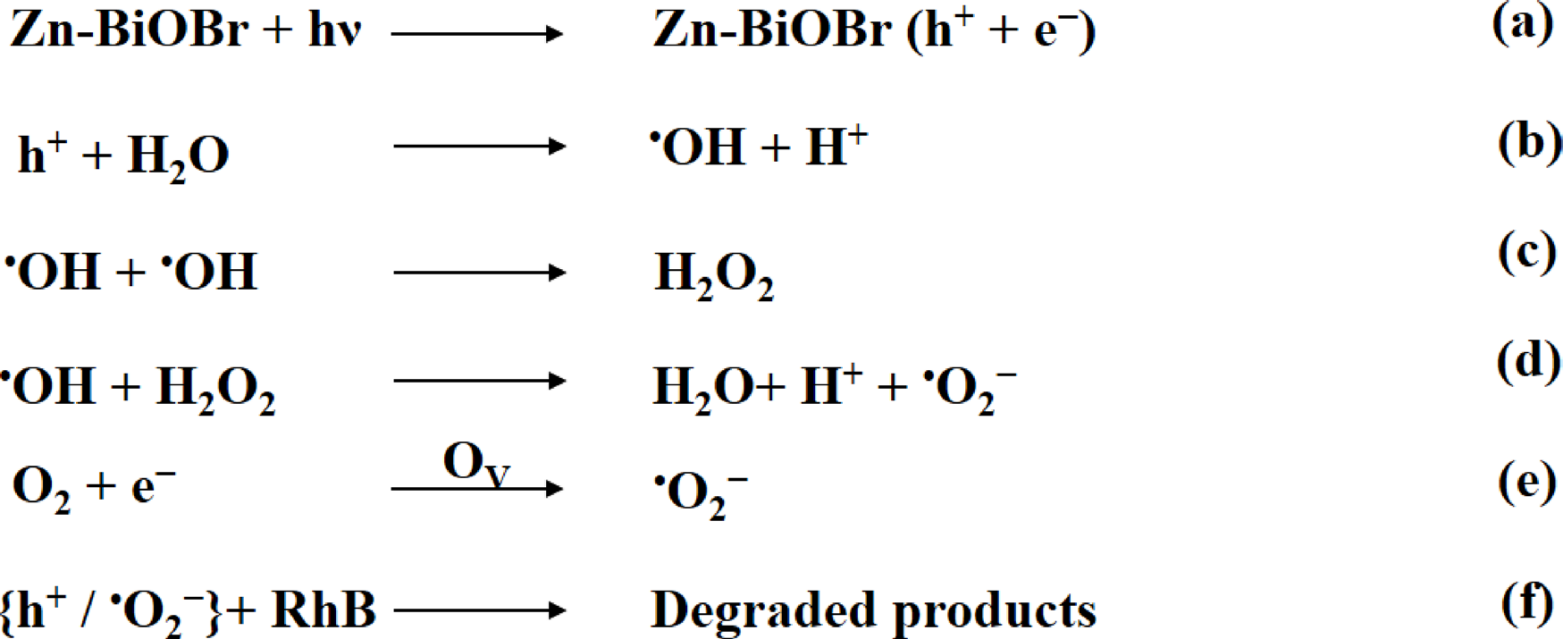
The active species generation over Zn-BiOBr and their role in RhB degradation.

The IPA promoted RhB photodegradation probably follows the same mechanism if the photogenerated ^•^OH radicals are induced by e^−^ from CBMs. Since the energy of ^•^OH radical is more positive than HOMO of RhB* but more negative than holes of photocatalysts VBMs, ^•^OH radicals can accept holes from VBMs but cannot accept h^+^ from HOMO of RhB*. However, because IPA improved RhB photodegradation on Zn-BiOBr, the IPA quenched ^•^OH radicals, which can consume more electrons and minimize their recombination to h^+^ at VBMs (Fig. 7)^15^. Scheme 1 provides a summary of active species generation and their role in RhB degradation.

### Photocatalytic activity of PVDF − Zn-BiOBr sponge

SEM and EDAX were used to investigate the sponge’s morphology and elemental analysis.

**Fig. 8:**
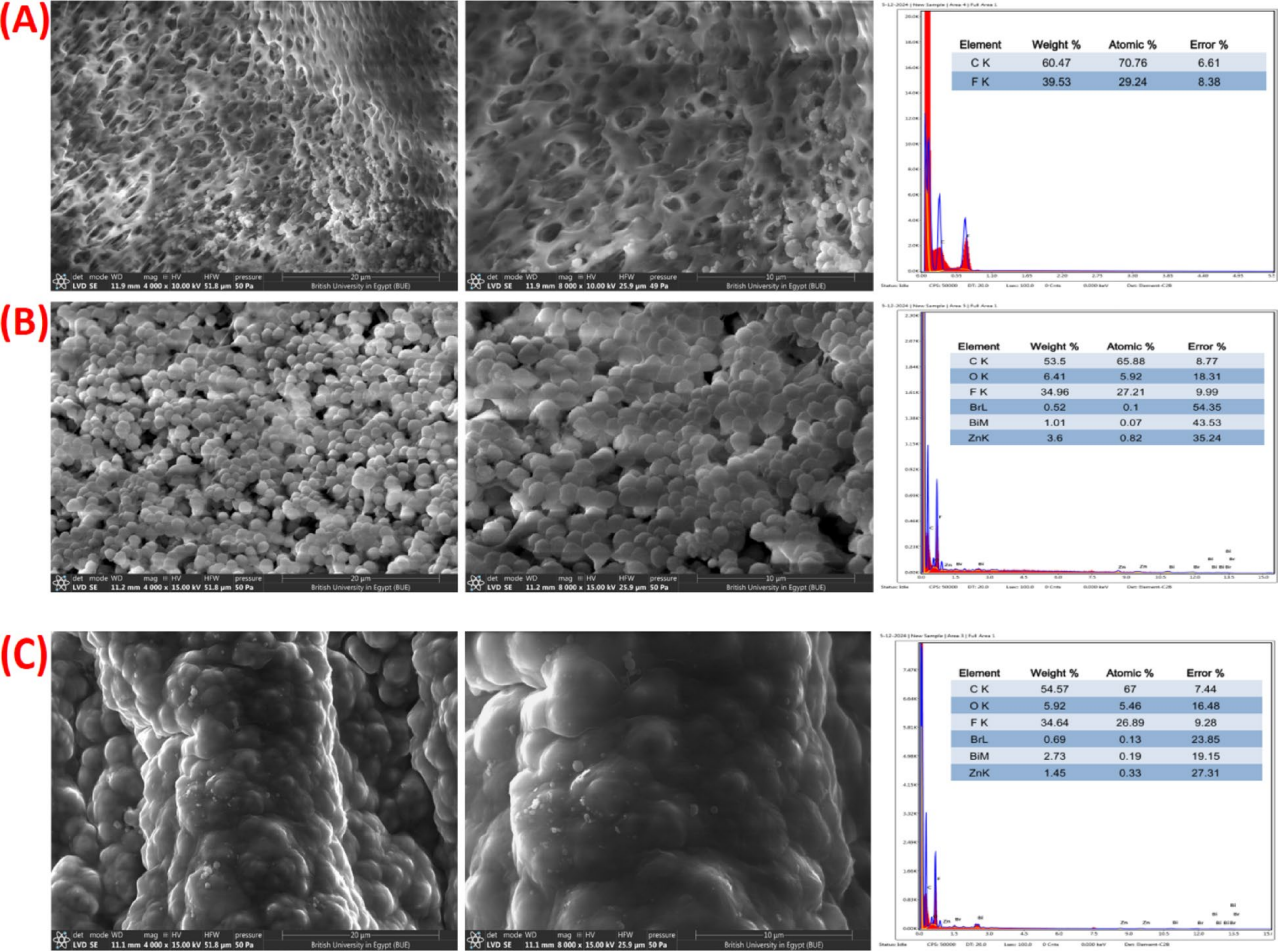
**(A)** The SEM images and corresponding EDAX of **(A)** the blank PVDF sponge **(B)** ZnBS-10, and **(C)** ZnBS-20.

Figure 8 shows SEM images of the cross section of the blank PVDF, ZnBS-10, and ZnBS-20 with corresponding EDAX spectrum. The pore structure of PVDF sponge has been shown by the SEM images Fig. 8A and anchoring of Zn-BiOBr in the pore interface of PVDF matrix was clearly observed **(**Fig. 8B**).** While, the porosity structure was blocked upon increasing the content of Zn-BiOBr (Fig. 8C). Furthermore, elemental composition of the prepared PVDF − Zn-BiOBr sponge was investigated by using EDAX analysis. Briefly, in Fig. 8A sponge composed with C and F while, ZnBS-10 and ZnBS-20 composed of C, F, O, Br, Bi, and Zn indicated intercalation of Zn-BiOBr into PVDF matrix furthermore as illustrated in Fig. 8B and Fig. 8C respectively.

**Fig. 9:**
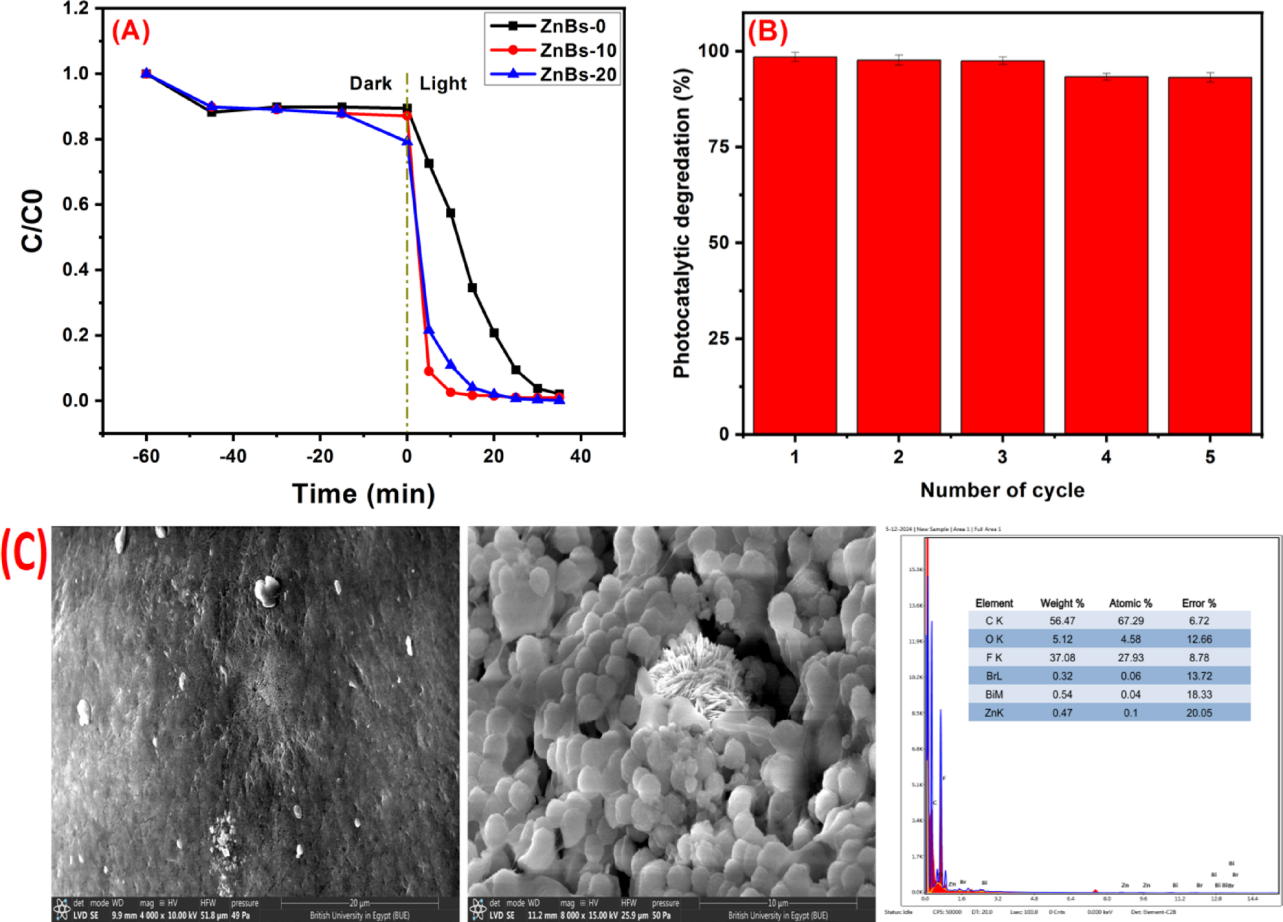
(**A**) The photocatalytic degradation of RhB with ZnBS-0, ZnBS-10 and ZnBS-20 under visible-light, (**B**) the photocatalytic durability of the as-fabricated ZnBS-10 sponge, and **(C)** SEM images and corresponding EDAX of ZnBS-10 after 5-cycle of RhB’s degradation.

As illustrated in Fig. 9A, the RhB concentration reduced under irradiation of visible light with using ZnBS0, ZnBS-10, and ZnBS-20 sponge. When compared to Zn-BiOBr powder, ZnBS-10’s activity is more robust and the dye removal achieved 100% after 15 min, While, the ZnBS-20 exhibited 65% dye removal at the same time that could be attributed to the reduction of porosity structure as illustrated by SEM images (Fig. 8C) and hence minimize the surface area of ZnBS-20. Generally, the enhancement of the dye removal upon via using PVDF based matrix could be attributed to the high adsorption ability of hydrophobic PVDF matrix^46,47^ and the nature promotion effect of spatial electric field of organic PVDF on charge separation rather than the particular properties of photocatalysts ^77^.

Additionally, the ZnBS-10 sponge can maintain its photocatalytic activity even after several reuses, according to the results of the recyclability test (Fig. 9B). The sponge demonstrated consistent activity for five runs in consecutive cycles as calculated in Table 2, suggesting that the manufactured sponge is a flexible 3D scaffold (dip-photocatalyst) could be removed from the reaction system easily (via forceps) and might be used for an extended period of time as a dip-photocatalyst without the requirement of challenging techniques to extract the photocatalyst in suspension from the reaction system. This finding further supports the strong conjugation of the Zn-BiOBr photocatalyst to the surface of PVDF matrix. These results are consistent with earlier studies in the field and support the efficiency and reliability of the proposed sponge-based photocatalytic system^46,47,52,77^. Furthermore, to assess the stability of the fabricated sponge SEM in addition to EDAX was performed (Fig. 9C.**).** As demonstrated, the matching EDAX spectrum is nearly identical before and after five consecutive cycles of RhB’s removal, and there are no discernible changes in the morphology of ZnBS-10 following these cycles, confirming the tremendous stability and durability of the fabricated sponge in addition to the easily removal and application in the reaction system after each cycle.

**Table 2:** The dye removal percentage for 5-cylces over ZnBS-10 sponge.

Number of cycle	Dye removal %	± SD
**1**	**99.5**	**1.2**
**2**	**98.7**	**1.3**
**3**	**98.5**	**0.98**
**4**	**94.3**	**0.89**
**5**	**94.2**	**1.2**

**Fig. 10:**
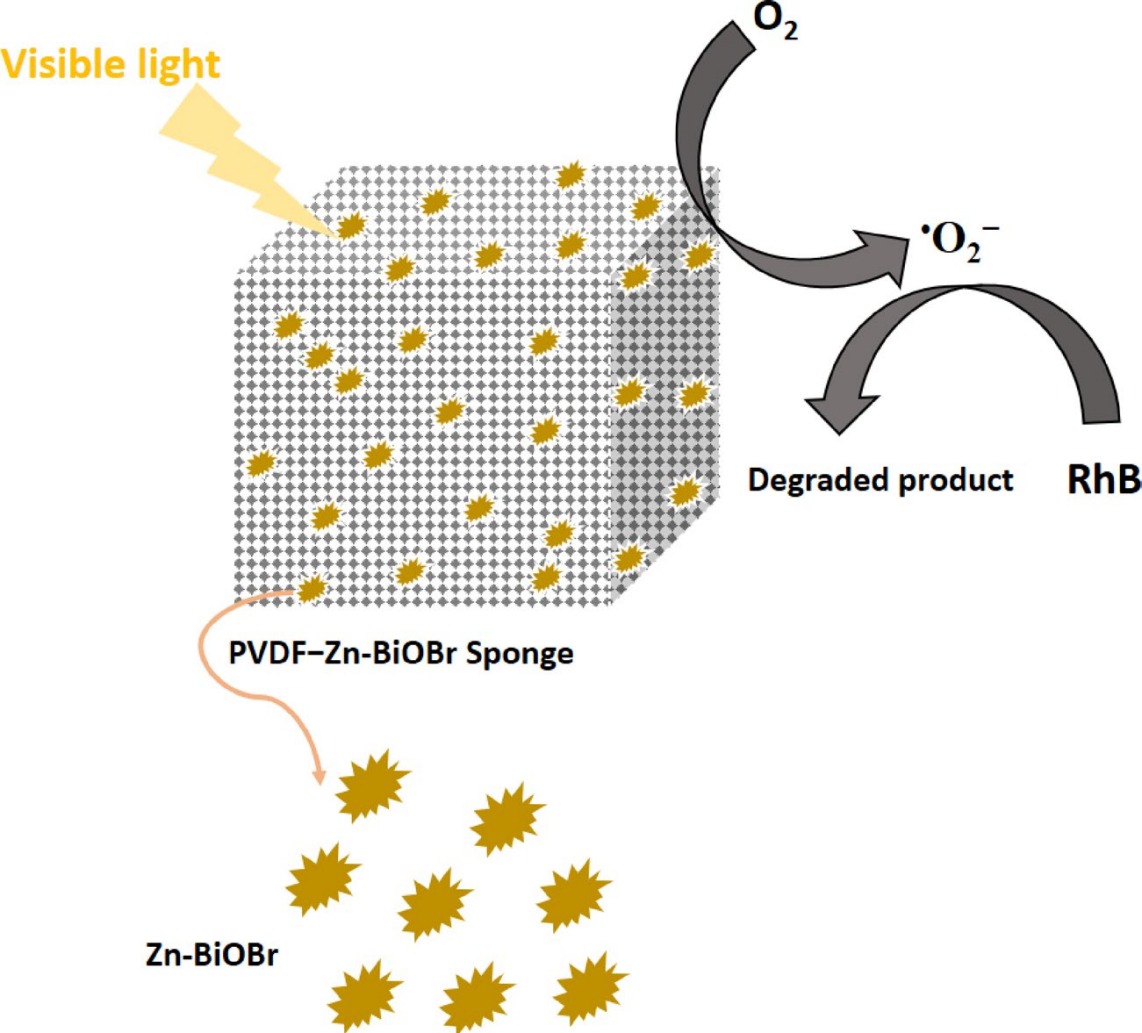
The proposed mechanism of the photo-catalytic degradation reaction over PVDF-Zn-BiOBr.

Based on the aforementioned findings and earlier research, the degradation reaction mechanism is proposed in Fig. 10. Because of PVDF’s porous structures’ high adsorption capacity, organic contaminants can be efficiently placed close to the Zn-BiOBr conjugated to the pore surface. then, Zn-BiOBr was excited via visible-light irradiation. After that, (I) photo generated electro-hole pair are formed. Highly reactive radical species can be generated as aforementioned (scheme 1) when the excited electrons react with the adsorbent molecular oxygen. Meanwhile, the h^+^ generate hydroxyl free radicals subsequently, The RhB pollutant is degraded by the ROS produced.

.

## Conclusion

The BiOBr and zinc doped BiOBr were successfully synthesized via solvo-thermal approach, with study the impact of zinc doping on the morphology, chemical composition, optical features, and Photocatalytic performance of BiOBr. Based on the obtained results, the zinc doping of BiOBr introduce O_V_ into the electronic configuration, stimulate strong synergistic effect between adjacent O_V_. doping induced lattice distortion excited O_V_ generation, which enhance carrier separation ability. Furthermore, a slightly shift of the absorption edge towards higher band-gap value, form 2.811 eV (pure BiOBr) to 2.831 eV (Zn-BiOBr) while, the PL spectrum of Zn-BiOBr was lower than BiOBr and the EIS results confirms the low resistance of charge transfer indicating electron–hole separation improvement upon doping of Zinc into BiOBr structure. Consequently, the photocatalytic activity was improved. High photocatalytic efficiency, sufficient dependability was demonstrated by the fabricated sponge due to the high adsorption ability of hydrophobic PVDF matrix and the nature promotion effect of spatial electric field of organic PVDF on charge separation rather than the particular properties of Zn-BiOBr. Furthermore, the fabricated sponge demonstrated tremendous stability and durability in addition to the easily removal and application in the reaction system. Based on its simple preparation method, photocatalytic activity and high reusability, the PVDF − Zn-BiOBr sponge is a promising material for energy conversion applications and environmental purposes and enables the reuse of the photocatalyst several times. Finally, accomplished results from this work are foreseen to contribute to the development of sustainable strategies in pollution control at industries and protection of water resources toward future generations.

## Data Availability

The datasets used and/or analysed during the current study available from the corresponding author on reasonable request.
